# Supplementary figures and images for: Induction of flight via midbrain projections to the cuneiform nucleus
Source: PLoS One. 2023 Feb 16;18(2):e0281464. doi: 10.1371/journal.pone.0281464 (PMC9934373; doi:10.1371/journal.pone.0281464)

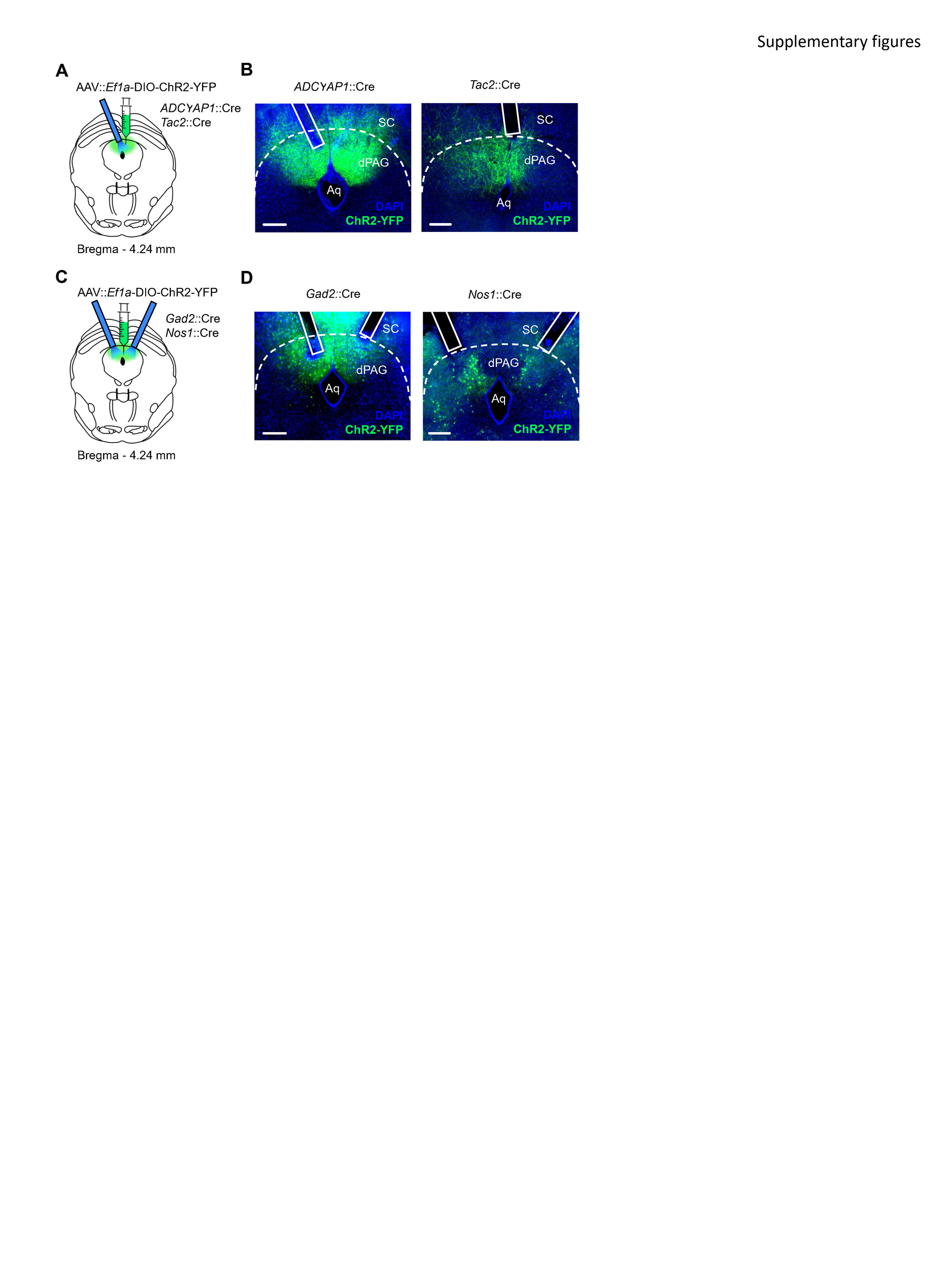

Supplement: S1 Fig — (A-C) Graphical representation of experimental strategy for optogenetic activation of cell-types in dPAG. (B-D) Representative histology showing ChR2 expression (green) and fibre placement for each Cre driver line (solid line; scale bar, 250 μm). (TIF) [file pone.0281464.s001.tif]

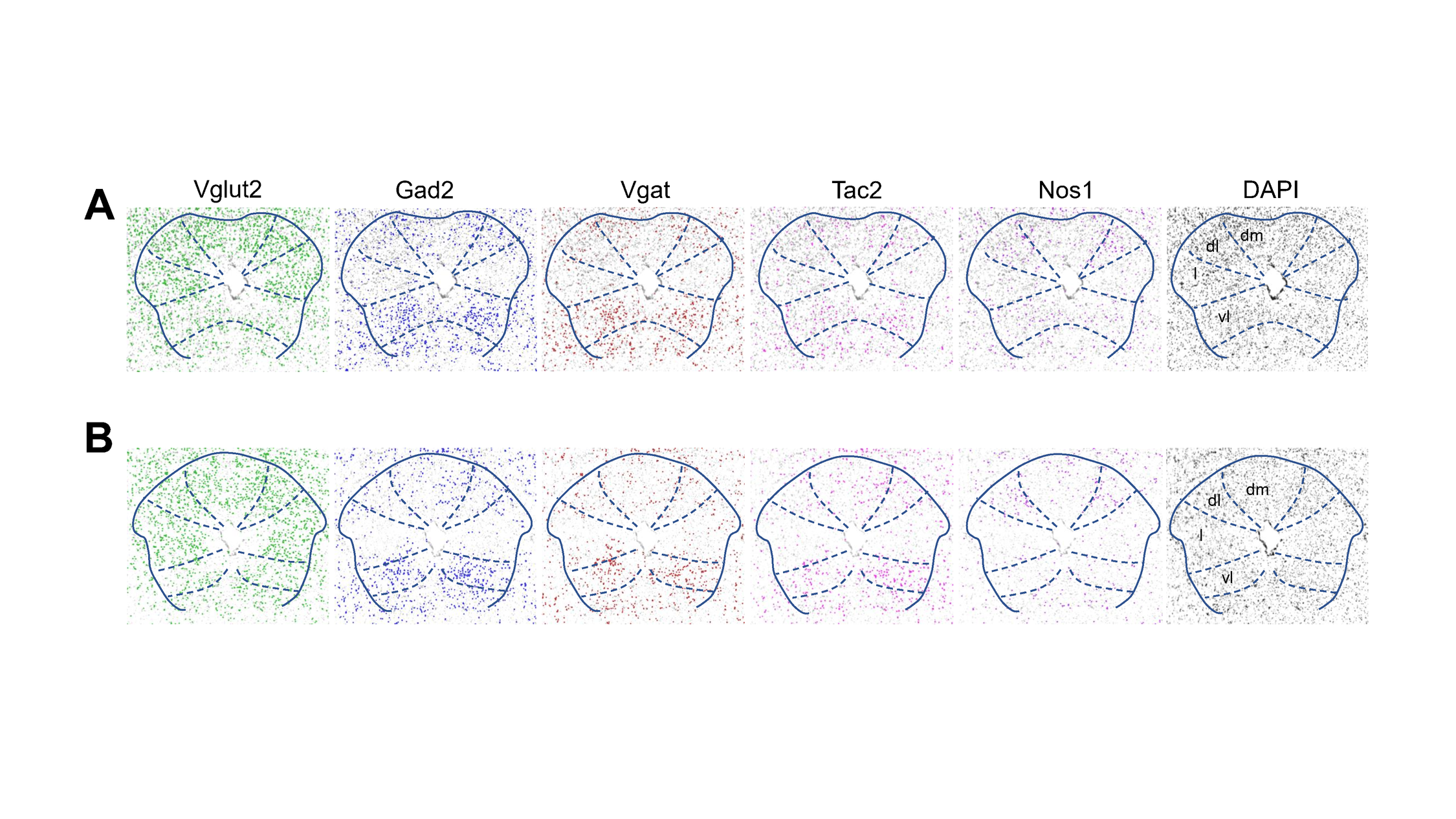

Supplement: S2 Fig — (A-B) Distribution of excitatory, inhibitory, Nos1 and Tac2 cells in PAG. Localization of Vglut2, Vgat, Gad2, Nos1, and Tac2 transcripts in PAG using multiplex in situ sequencing in two independent brain sections. (TIF) [file pone.0281464.s002.tif]

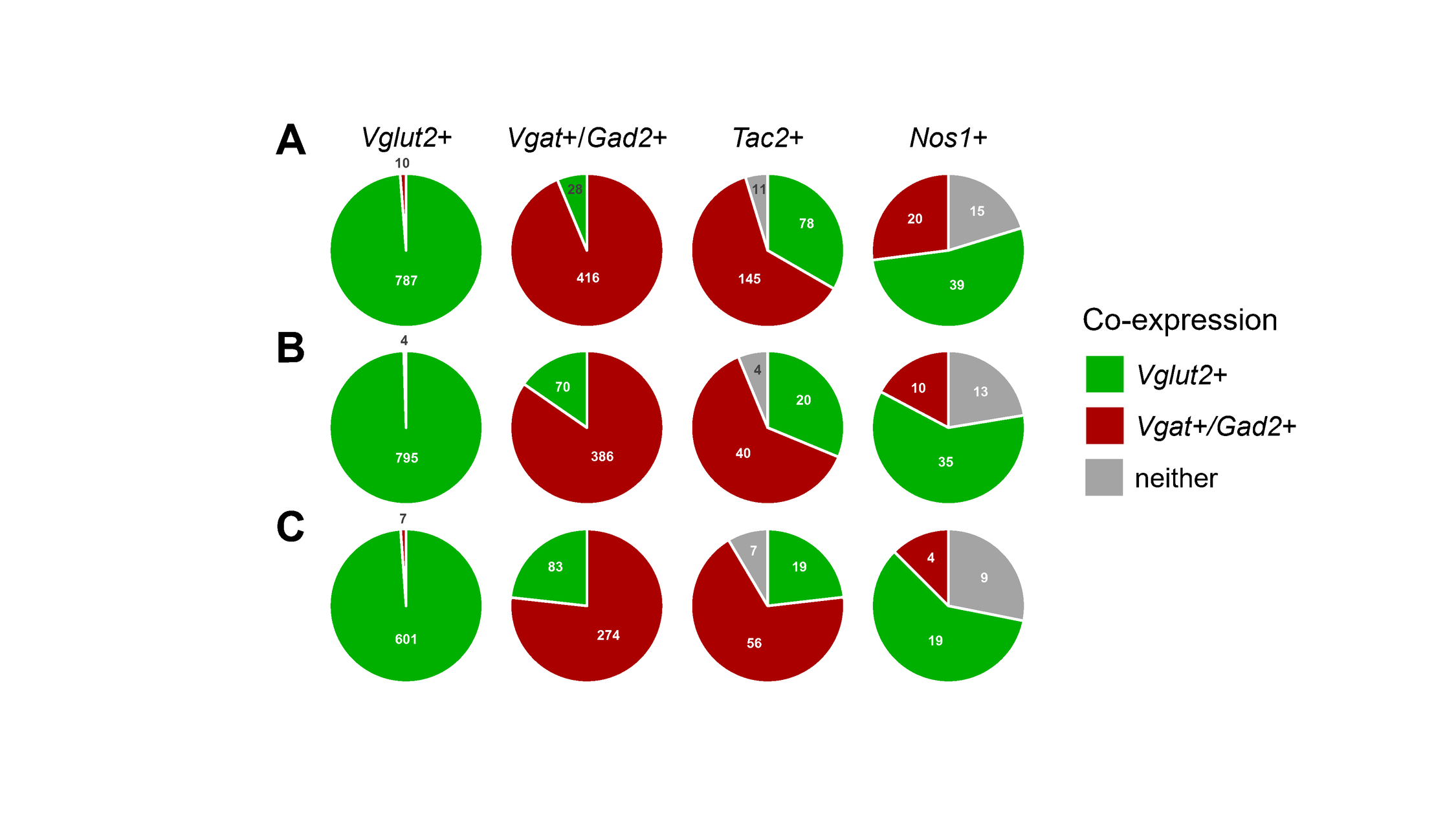

Supplement: S3 Fig — (A-C) Distribution of co-expression of glutamatergic and GABAergic markers and their co-localization with Nos1 and Tac2 in three independent brain sections. (TIF) [file pone.0281464.s003.tif]
